# A Convenient, TiCl_**4**_/SnCl_**4**_-Mediated Synthesis of *N*-Phenyl or *N*-Aryl Benzamidines and *N*-Phenylpicolinamidines

**DOI:** 10.5402/2012/963195

**Published:** 2012-07-29

**Authors:** Umesh D. Patil, Pramod P. Mahulikar

**Affiliations:** School of Chemical Sciences, North Maharashtra University, Jalgaon 425 001, India

## Abstract

A new, TiCl_4_-or SnCl_4_-mediated, solvent-free method was developed for the synthesis of *N*-Aryl benzamidines and *N*-phenylpicolinamidines, in moderate-to-good yield, using suitable amines and nitriles as starting materials.

## 1. Introduction

The amidine nucleus is found in a wide variety of biologically active molecules [1]. *N*-Aryl amidine exhibits activity against *Mycobacterium tuberculosis, *and *N*-alkylfuramidine shows antiprotozoan and antimicrobial activities [2]. Similarly various amidines derived from 4-amidino-2-(2-pyridyl)quinazoline [3] and 1-amino-3-(2-pyridyl)isoquinoline [3], guanidine [4], diguanidino [5], reversed diamidino 2,5-diarylfuran [5], benzimidazole [6], pyridine [7], exhibit antimycoplasmal, antimalarial, antimicrobial, antibacterial, anti-inflammatory activities. An extensive number of monoamidines have been evaluated for their utility in blocking various stages of the thrombin cascade, and numerous highly potent molecules have been reported [8].

Amidines were used as important synthon in organic synthesis in the preparation of various heterocyclic compounds, such as pyridine [7, 9], pyrimidines [9, 10], imidazoles [11], pyrazolopyrimidine [12], iminopyrimidine [13], imidazopyridine and pyrimidinopyridine [14], purine [15], benzimidazole [16], pyrimidines [17], triazaphenalene [18], triazine [19], tetraazole [19], thiadiazine [20], oxazolotriazole [21], diazirine [22], triazolopyridine [23], azetidinone [24], and pyrrole, and also used as complexing agent [25].

Several synthetic strategies have been developed for the synthesis of amidines, in which the nucleophilic addition of amine to nitrile is the most popular. Generally, nitriles were activated to the intermediate salt in the presence of EtOH/HCl [26] or NH_4_Cl/MeOH [27] under anhydrous condition and then reacted with amine to get amidine. While for unreactive nitriles, Lewis acid or other condensing agents were used such as anhydrous AlCl_3_, ZnCl_2_ [28], CuCl [29], Ln (III) salts [30], CaCl_2 _[31], Al(CH_3_)_3_ [32], SmI_2_ [33], Ytterbium amide [34], MeSO_3_H [20], and anhyd. SnCl_4_ [21]. Amides can be converted to imidoyl chloride using PCl_5_ [35, 36], which can then react with primary or secondary amine to yield amidine. In addition, amide can be O-alkylated with triethyloxonium fluoroborate at ambient temperature to yield the corresponding imidic ester fluoroborate, which then reacts with amine to yield the targeted amidine [3]. Iron pentacarbonyl was employed to the conversion of amidoximes into amidines via reductive cleavage of the N=O bond [37]. Sometimes, strong bases like LiHMDS, NaHMDS, LDA, BuLi, NaOMe [38], and NaH [20] were used as condensing agent. Similarly, Dains F. B. has shown that amidine was prepared from symmetrical diaryl and dialkyl urea and acid chloride [39, 40].

In 1998, Zhou and Zhang published the results on such a subject that amidines were successfully prepared from nitriles and nitrocompounds in the presence of TiCl_4_/Sm in THF. They also reported that under same reaction conditions amidine formation was not observed by treatment of nitriles with amines [41]. Thus, it was of interest to study the reactions of nitrile and amine using TiCl_4_ and SnCl_4_.

## 2. Results and Discussion

**Scheme 1:**
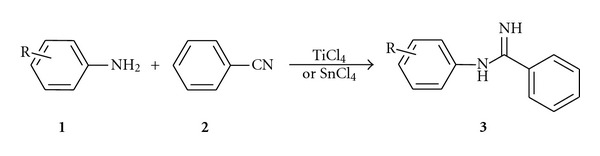


We would like to demonstrate in the present work that amidine could be prepared by coupling nitrile with amine in presence of TiCl_4_ as well as SnCl_4_ using neat condition in absence of samarium. At the beginning we studied the synthesis of amidine (Scheme 1, Table 1, and Entry 1) using benzonitrile and aniline as model substrates. In a typical experiment aniline (0.01 mol) and benzonitrile (0.01 mol) were heated at 100–110°C with TiCl_4_ or SnCl_4_ (0.012 mol) for 3-4 hr to complete the reaction. The obtained black reaction mixture was then neutralised with NaOH solution and extracted with dichloromethane. Product was isolated simply by evaporation of the solvent at reduced pressure. The crude product was recrystallized from hexane. The obtained product was characterised by IR, NMR, and mass spectroscopy data and compared with authentic sample. Furthermore, the reaction was carried out for several substituted aryl amines and nitriles (Table 1) under the same conditions. It is distinct that both TiCl_4_ and SnCl_4_ were found to have a potential utility for the synthesis of amidine with good-to-moderate yields under mentioned reaction conditions.

**Scheme 2:**
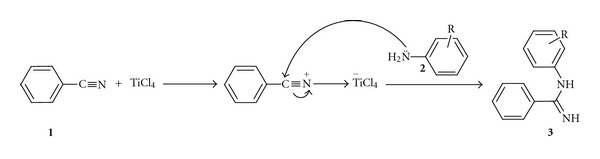
Mechanism.

The mechanism we propose for the reaction is similar to the one reported for amidine using AlCl_3_ [42] and is outlined in Scheme 2: at first the complex between nitrile and TiCl_4_ was formed followed by nucleophilic addition of amine (**2**) on the more electrophilic carbon of nitriles to yield the amidine (**3**). 

**Scheme 3:**
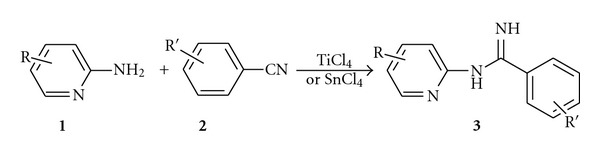


With these results in hand, we tested the scope and limitations of this process; we examined the coupling reaction of various substituted benzonitriles with heteroaromatic amine, that is, 2-aminopyridine, (Scheme 3 and Table 2) by performing the reaction with the well-established reaction conditions.

**Scheme 4:**
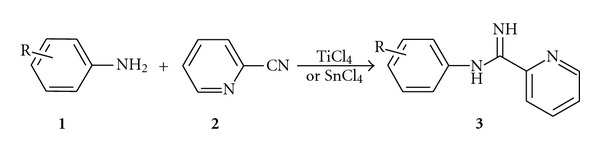


Similarly, to test the scope and limitations of this process; we examined the coupling reaction of various substituted anilines with heteroaromatic nitrile, that is, 2-cyanopyridine, (Scheme 4 and Table 3) by performing the reaction with the well-established reaction conditions.

## 3. Conclusion

In the summary, we have developed a solvent-free method of amidine formation from nitrile and amine using TiCl_4_ or SnCl_4_ in absence of expensive metal-like samarium. The reaction proceeded at 100–110°C and was completed within 3-4 hrs. In conclusion the reaction was extremely simple to carry out, and the obtained yield of amidine was good to moderate. On the basis of yield, we may conclude that TiCl_4_ is preferable catalyst over SnCl_4_.

## 4. Experimental

Melting points were determined by open capillary tube method and are uncorrected. Progress of the reaction was monitored by TLC (visualization was effected by exposure to UV light). Commercial reagents were used without purification for synthesis. Mass spectra were recorded on Thermo Finnigan (model- LCQ Advantage MAX) mass spectrometer. The IR spectra were recorded with KBr pellets on Perkin-Elmer Spectrum One Spectrometer. ^1^H NMR spectra were recorded in CDCl_3_ on a Bruker 300 DRX Avance instrument at 300 MHz.

### 4.1. Preparation of Amidines, **3a–o**

Benzonitrile (1.03 g, 0.01 mol) was taken in a dry round bottom flask and to this was added a 2-aminopyridine (0.94 g, 0.01 mol). The flask was heated, after fitting a dry condenser along with a guard tube, in an oil bath at a temperature range of 80–90°C with stirring. After 30 min TiCl_4_ (1.3 mL, 0.012 mol) or SnCl_4_ (1.4 mL, 0.012 mol) was added to the flask. After addition, temperature was increased to 100–110°C, and contents of the flask were heated for 3-4 hrs. The mixture was cooled to room temperature, and the solid, thus, formed was dissolved in hot water and made alkaline with 10% NaOH. This solution was extracted with a CH_2_Cl_2_  (3 × 100 mL).  Then organic layer was decolourized with activated charcoal and dried over anhydrous Na_2_SO_4_. After evaporating the solvent under reduced pressure, crude amidine was obtained. This crude product was recrystallized from hexane to get pure amidine.

**N-Phenylbenzamidine (3a, C_13_H_12_N_2_)**

^1^H NMR (CDCl_3_, *δ* ppm): 4.84 (br s, 2H, NH, C=NH), 6.96–6.99 (d, J=8.1 Hz, 2H, ArH), 7.03–7.08 (t, J=7.5 Hz 1H, ArH), 7.32–7.37 (t, J=7.8 Hz, 2H, ArH), 7.42–7.49 (m, 3H, ArH), 7.85 (s, 2H, ArH); IR (KBr) *ν* (cm^−1^): 3340, 2853, 1618, 1574, 1459, 1377, 1153, 722; MS (ESI, 70 eV) *m/z* (%): 198 (13), 197 (100) [M + H]^+^.

**N-(2-Chlorophenyl)benzamidine (3b, C_13_H_11_ClN_2_)**

^1^H NMR (CDCl_3_, *δ* ppm): 4.78 (br s, 2H, NH, C=NH), 6.97–7.02 (m, 2H, ArH), 7.19–7.22 (t, J=7.2 Hz,1H, ArH), 7.39–7.51 (m, 4H, ArH), 7.86 (br s, 2H, ArH); IR (KBr) *ν* (cm^−1^): 3345, 2853, 1618, 1574, 1459, 1377, 1153, 722; MS (ESI, 70 eV) *m/z* (%): 233 (37), 231(100) [M + H]^+^.

**N-(4-Fluorophenyl)benzamidine (3c, C_13_H_11_FN_2_)**

^1^H NMR (CDCl_3_, *δ* ppm): 4.86 (br s, 2H, 2NH), 6.90–6.94 (m, 2H, ArH), 7.01–7.07 (t, J=8.3 Hz, 2H, ArH), 7.41–7.46 (t, J=7.3 Hz, 3H, ArH), 7.83–7.86 (d, J=6.3 Hz, 2H, ArH); IR (KBr) *ν* (cm^−1^): 3348, 2854, 1625, 1590, 1459, 1377, 1152, 777, 722; MS (ESI, 70 eV) *m/z* (%): 216 (17), 215 (100) [M + H]^+^, 198 (13).

**N-(Pyridin-2-yl)benzamidine (3d, C_12_H_11_N_3_)**

^1^H NMR (CDCl_3_, *δ* ppm): 2.03 (bs, 2H, 2NH), 6.90–6.95 (m, 1H, ArH), 7.25–7.29 (d, J=9 Hz, 1H, ArH), 7.40–7.48 (m, 3H, ArH), 7.61–7.67 (m, 1H, ArH), 7.89–7.92 (m, 2H, ArH), 8.31–8.34 (m, 1H, ArH); IR (KBr) *ν* (cm^−1^): 3351, 2854, 1625, 1590, 1459, 1377, 1152, 777, 722; MS (ESI, 70 eV) *m/z* (%): 198 (100) [M + H]^+^.

**3-Chloro-N-(pyridin-2-yl)benzamidine (3e, C_12_H_10_ClN_3_)**

^1^H NMR (CDCl_3_, *δ* ppm): 2.03 (bs, 2H, 2NH), 6.92–6.96 (m, 1H, ArH), 7.25–7.45 (m, 3H, ArH), 7.62–7.68 (m, 1H, ArH), 7.72–7.73 (d, J=1.5 Hz, 1H, ArH), 7.92–7.93 (t, J=1.8 Hz, 1H, ArH), 8.32–8.34 (m, 1H, ArH); IR (KBr) *ν* (cm^−1^): 3341, 2854, 1625, 1590, 1459, 1377, 1152, 777, 722; MS (ESI, 70 eV) *m/z* (%): 231 (100) [M + H]^+^.

**4-Chloro-N-(pyridin-2-yl)benzamidine (3f, C_12_H_10_ClN_3_)**

^1^H NMR (CDCl_3_, *δ* ppm): 1.81 (br s, 2H, 2NH), 6.93–6.97 (m, 1H, ArH), 7.25–7.27 (t, J=8.1 Hz,1H, ArH), 7.41–7.43 (d, J=6.3 Hz, 2H, ArH), 7.63–7.69 (m, 1H, ArH), 7.85–7.89 (d, J=6.9 Hz, 2H, ArH), 8.33–8.35 (m, 1H, ArH); IR (KBr) *ν* (cm^−1^): 3344, 2853, 1618, 1594, 1462, 1377, 1125, 831, 782, 722, 538; MS (ESI, 70 eV) *m/z* (%): 231 (100) [M + H]^+^.

**4-Bromo-N-(pyridin-2-yl)benzamidine (3g, C_12_H_10_BrN_3_)**

^1^H NMR (CDCl_3_, *δ* ppm): 1.78 (br s, 2H, 2NH, C=NH), 6.92–6.97 (m, 1H, ArH), 7.24–7.27 (t, J=3.9 Hz,1H, ArH), 7.55–7.60 (m, 2H, ArH), 7.63–7.69 (m, 1H, ArH), 7.72–7.81 (m, 2H, ArH), 8.32–8.35 (m, 1H, ArH); IR (KBr) *ν* (cm^−1^): 3349, 2854, 1640, 1577, 1530, 1459, 1377, 1321, 1300, 1260, 1151, 1119, 1096, 1006, 818, 769, 722, 541; MS (ESI, 70 eV) *m/z* (%): 278 (100), 276 (100) [M + H]^+^.

**N-(5-Bromopyridin-2-yl)benzamidine (3h, C_12_H_10_BrN_3_)**

^1^H NMR (CDCl_3_, *δ* ppm): 1.68 (br s, 2H, NH), 7.16–7.19 (d, 1H, ArH), 7.42–7.49 (m, 3H, ArH), 7.71–7.75 (m, 1H, ArH), 7.88–7.91 (t, J=6 Hz, 2H, ArH), 8.37–8.38 (d, J=2.4 Hz, 1H, ArH); IR (KBr) *ν* (cm^−1^): 3350, 2853, 1618, 1574, 1459, 1377, 1153, 722; MS (ESI, 70 eV) *m/z* (%): 278 (94), 276 (100) [M + H]^+^, 260 (90), 259 (90).

**N-Phenylpicolinamidine (3i, C_12_H_11_N_3_)**

^1^H NMR (CDCl_3_, *δ* ppm): 5.91 (br s, 2H, NH, C=NH), 6.88–7.26 (m, 4H, ArH), 7.31–7.37 (m, 2H, ArH), 7.78–7.81 (m, 1H, ArH), 8.41–8.45 (m, 1H, ArH), 8.52–8.59 (m, 1H, ArH); IR (KBr) *ν* (cm^−1^): 3346, 2853, 1618, 1574, 1459, 1377, 1153, 722; MS (ESI, 70 eV) *m/z* (%): 198 (100) [M + H]^+^, 181 (45).

**N-o-tolylpicolinamidine (3j, C_13_H_13_N_3_)**

^1^H NMR (CDCl_3_, *δ* ppm): 2.18 (s, 3H, ArCH_3_), 5.72 (br s, 2H, NH, C=NH), 6.89–7.02 (m, 2H, ArH), 7.16–7.24 (q, J=7.8 Hz, 1H, ArH), 7.30–7.33 (dd, J=8.4 Hz,1H, ArH), 7.36–7.41 (m, 1H, ArH), 7.77–7.84 (m, 1H, ArH), 8.36–8.48 (m, 1H, ArH), 8.55–8.57 (m, 1H, ArH); IR (KBr) *ν* (cm^−1^): 3345, 1694, 1613, 1463, 1377, 1118, 721; MS (ESI, 70 eV) *m/z* (%): 212 (100) [M + H]^+^, 195 (37).

**N-p-tolylpicolinamidine (3k, C_13_H_13_N_3_)**

^1^H NMR (CDCl_3_, *δ* ppm): 2.18 (s, 3H, ArCH_3_), 5.58 (br s, 2H, NH, C=NH), 6.90–7.01 (m, 3H, ArH), 7.18–7.26 (m, 2H, ArH), 7.36–7.40 (m, 1H, ArH), 7.56 (m, 1H, ArH), 7.77–7.84 (m, 1H, ArH); IR (KBr) *ν* (cm^−1^): 3339, 2854, 1694, 1613, 1463, 1377, 1118, 721; MS (ESI, 70 eV) *m/z* (%): 212 (100) [M + H]^+^, 195 (37).

**N-(4-Fluorophenyl)picolinamidine (3l, C_12_H_10_FN_3_)**

^1^H NMR (CDCl_3_, *δ* ppm): 1.76 (br s, 1H, NH), 5.81 (br s, 1H, NH), 6.88–7.02 (m, 4H, ArH), 7.32–7.35 (m, 1H, ArH), 7.73–7.78 (m, 1H, ArH), 8.32–8.34 (d, J=6 Hz, 1H, ArH), 8.49–8.50 (d, J=6 Hz, 1H, ArH); IR (KBr) *ν* (cm^−1^): 3378, 2854, 1625, 1590, 1459,1377, 1152, 777, 722; MS (ESI, 70 eV) *m/z* (%): 216 (100) [M + H]^+^, 199 (25).

**N-(4-Chlorophenyl)picolinamidine (3m, C_12_H_10_ClN_3_)**

^1^H NMR (CDCl_3_, *δ* ppm): 5.86 (br s, 2H, NH, C=NH), 6.84–6.88 (m, 2H, ArH), 7.25–7.42 (m, 3H, ArH), 7.73–7.82 (t, J=5.7 Hz, 1H, ArH), 8.29–8.40 (m, 1H, ArH), 8.51–8.59 (m, 1H, ArH); IR (KBr) *ν* (cm^−1^): 3345, 2853, 1618, 1574, 1459, 1377, 1153, 722; MS (ESI, 70 eV) *m/z* (%): 232 (100) [M + H]^+^, 215 (33).

**N-(4-Bromophenyl)picolinamidine (3n, C_12_H_10_BrN_3_)**

^1^H NMR (CDCl_3_, *δ* ppm): 5.85 (br s, 2H, NH, C=NH), 6.88–6.91 (d, J=8.7 Hz, 2H, ArH), 7.38–7.50 (m, 3H, ArH), 7.79–7.84 (m, 1H, ArH), 8.36–8.39 (d, J=8.1 Hz, 1H, ArH), 8.56–8.58 (m, 1H, ArH); IR (KBr) *ν* (cm^−1^): 3376, 2854, 1640, 1577, 1530, 1459, 1377, 1321, 1300, 1260, 1151, 1119, 1096, 1006, 818, 769, 722, 541; MS (ESI, 70 eV) *m/z* (%): 278 (100), 276 (100) [M + H]^+^, 260 (20), 259 (20).

**N-(3,4-Dichlorophenyl)picolinamidine (3o, C_12_H_9_Cl_2_N_3_)**

^1^H NMR (CDCl_3_, *δ* ppm): 5.97 (br s, 2H, 2NH), 6.84–6.88 (m, 1H, ArH), 7.12–7.17 (t, 1H, ArH), 7.39–7.43 (t, J=3.9 Hz,2H, ArH), 7.79–7.87 (t, J=9.6 Hz, 1H, ArH), 8.33–8.36 (d, J=7.8 Hz,1H, ArH), 8.56–8.58 (d, J=4.5 Hz, 1H, ArH); IR (KBr) *ν* (cm^−1^): 3345, 1694, 1613, 1463, 1377, 1118, 721; MS (ESI, 70 eV) *m/z* (%): 267 (100), 266(65) [M + H]^+^.

## Figures and Tables

**Table 1:** SnCl_4_/TiCl_4_ catalysed coupling of substituted anilines with benzonitrile.

Entry no.	Amines	Amidines^a^ (isolated yield %)		M.P. ^°^C (Lit.)
		by TiCl_4_	by SnCl_4_
**3a**	Aniline	75	71	114–116 (116)
**3b**	2-Cl-Aniline	65	67	108–110
**3c**	4-F-Aniline	64	63	86–88

^a^All products were characterised by IR, NMR, and mass spectral data and in comparison with authentic samples.

**Table 2:** SnCl_4_/TiCl_4_ catalysed coupling of 2-aminopyridine with substituted benzonitriles.

Entry no.	Amines		Nitriles	Amidines^a^ (isolated yield %)		M.P.^°^C (Lit.)
	R	X	R^′^	by TiCl_4_	by SnCl_4_
**3d**	H	N	H	72	61	96 (97-98)
**3e**	H	N	3-Cl	63	68	114–116
**3f**	H	N	4-Cl	69	63	162–164
**3g**	H	N	4-Br	73	66	152–154
**3h**	4-Br	N	H	67	63	102–104

^a^All products were characterised by IR, NMR, and mass spectral data and in comparison with authentic samples.

**Table 3:** SnCl_4_/TiCl_4_ catalysed coupling of substituted anilines with 2-cyanopyridine.

Entry no.	Amines	Amidines^a^ (isolated yield %)		M.P. ^°^C (Lit.)
	R	by TiCl_4_	by SnCl_4_
**3i**	H	79	68	78–80 HCl Salt
**3j**	2-CH_3_	59	64	68–70 (68-69)
**3k**	4-CH_3_	64	61	54 (52-53)
**3l**	4-F	78	69	72 (75-76)
**3m**	4-Cl	66	60	80–82 (80–82)
**3n**	4-Br	81	65	84–86 (85-86)
**3o**	3,4-Cl	76	69	112 (112-113)

^a^All products were characterised by IR, NMR, and mass spectral data and in comparison with authentic samples.
